# Prevalence of human papillomavirus types in head and neck cancer sub-sites in the Indian population

**DOI:** 10.3332/ecancer.2022.1358

**Published:** 2022-02-18

**Authors:** Devyani Gholap, Sharayu Mhatre, Pankaj Chaturvedi, Sudhir Nair, Tarik Gheit, Massimo Tommasino, Rajesh Dikshit

**Affiliations:** 1 Centre for Cancer Epidemiology, Tata Memorial Centre, Kharghar, Navi Mumbai 410210, India; 2 Homi Bhabha National Institute, Mumbai 400094, India; 3 Head and Neck Oncology, Department of Surgical Oncology, Tata Memorial Hospital, Mumbai 400012, India; 4 Infections and Cancer Biology Group, International Agency for Research on Cancer (IARC), 150 Cours Albert Thomas, 69372 Lyon, CEDEX 08, France; a https://orcid.org/0000-0001-6406-4562; b https://orcid.org/0000-0003-4830-0486

**Keywords:** human papillomaviruses, India, head and neck cancer, prevalence

## Abstract

Although a subset of head and neck cancers (HNC) has been associated worldwide with mucosal high-risk human papillomaviruses (HPV), information on the prevalence of HPV-positive HNC in India is limited. In this study, we examined the prevalence of 21 subtypes of HPV in sub-sites of HNC (*n* = 175) in the western region of India. Type-specific multiplex genotyping assay was conducted at the Centre for Cancer Epidemiology, Tata Memorial Centre, to determine the prevalence of HPV subtypes. The HPV prevalence was observed to be 28.43%, 41.67%, 38.89% and 15.79% in the oral cavity, oropharynx, hypopharynx and larynx tumour tissues, respectively. The HPV 16 genotype was most common in all HNC tumour tissues (30.29%), followed by HPV 58 (0.57%).

## Introduction

Head and neck cancer (HNC) includes a heterogeneous group of tumours occurring at different anatomical sites, e.g., oral cavity (OC), oropharynx (OPC), larynx (LC) and hypopharynx cancers (HPC). The age-standardised incidence rate of HNC attributed to human papillomaviruses (HPV) infection was 0.5–0.75 per 100,000 and >1.25 in India and Europe and US, respectively in 2012 [1]. Over the past decades, it is evident that a subset of HNC, e.g. OPC, is associated with certain mucosal high-risk (HR) HPV genotypes. HPV16 is responsible for majority of the HPV-positive HNCs, while the remaining HR HPV types appear to play a marginal role [2]. However, it is not yet clear whether the prevalence of HNC positive for mucosal HR HPV types other than HPV16 varies in different geographical areas. There are few studies on the Indian population that have studied the prevalence of low risk (LR) and HR HPV genotypes in all HNC subtypes using a standardised methodology. Most of the Indian studies have used polymerase chain reaction (PCR)-based amplification techniques using consensus or broad-spectrum primers to detect HPV. However, the use of such primers might underrepresent the true HPV prevalence, especially the prevalence of multiple infections. Also, in case of multiple infections, there is competition for amplification between primers if one HPV type is present in excess. This problem can be solved by using HPV type-specific primers. The E7 type-specific multiplex genotyping assay (E7-MPG) combines the multiplex polymerase chain reaction using specific HPV primers and hybridisation to type-specific oligonucleotide probes which allow us to determine the true HPV prevalence. Overall, the E7-MPG assay is much faster, comparatively economical and a less laborious means of HPV genotyping for epidemiological studies. In this study, we used E7-MPG assay, a simple, bead-based high throughput hybridisation method that allows the detection of 21 HPV genotypes (HR 16, 18, 26, 31, 33, 35, 39, 45, 51, 52, 53, 56, 58, 59, 66, 68, 73 and 82 and LR 6, 11 and 70) developed by Schmitt *et al* [3]. The aim of this study was to assess the prevalence of 21 HPV genotypes in the Indian population accompanied by studying the demographic characteristics.

## Materials and methods

The study was conducted at the Centre for Cancer Epidemiology, Mumbai, India. The HNC subtypes included in the study were OC (C00: Lip; C03: Gum; C04: Floor of mouth; C05: Palate; C06: Other; and unspecified parts of mouth), OPC (C01: Base of tongue; C09: Tonsil; and C10: Oropharynx), HPC (C12: Pyriform sinus and C13: Hypopharynx) and LC (C32) in the visiting outpatient department of Tata Memorial Hospital. Cases were recruited in the study from 2014 to 2017 only when they were histologically reported as an invasive tumour of the OC, OPC, HPC and LC. Primary cases aged 18–89 with the date of diagnosis not more than 6 months from date of interview were enrolled in the study by trained social investigators. Information of lifestyle habits such as tobacco smoking, chewing and alcohol drinking was collected on a standardised questionnaire.

Fresh head and neck tumour tissue biopsy or surgically resected specimens were collected in tissue stabilisation agent RNAlater®. The tissues were stored overnight at 4°C and processed the next day for DNA extraction by using Qiagen® DNAeasy Blood and Tissue kit as per the manufacturer’s instructions. After assessing the quantity and quality of the DNA products, they were then subjected to primer-specific multiplex amplification using Qiagen® multiplex PCR kit. Two primers for the amplification of β-globin were added to provide a positive control for the quality of the template DNA. The development of primers and its sequences have been described elsewhere [4]. To avoid cross contamination, quality control measures were taken, viz. PCR master mix preparation laminar air flow hood (LAF) and final DNA addition LAF were kept physically separate; PCR strip tubes with caps were used; and DNAse/RNAse free water was used as negative control for every PCR reaction.

After PCR amplification, the samples were analysed by E7-MPG using Luminex technology (Luminex Corporation, Austin, TX) as described elsewhere [5]. The HPV genotypes screened in the study were HPV16, HPV18, HPV45, HPV51, HPV35, HPV58, HPV39, HPV68, HPV56, HPV66, HPV59, HPV33, HPV52, HPV31, HPV26, HPV53, HPV70, HPV73 and HPV82. As part of quality control, intra-assay and inter-assay validation checks were performed. Tris-ethylenediaminetetraacetic acid buffer was used as a blank and PCR negative control was run as Luminex negative control. For each probe, the median fluorescence intensity (MFI) values obtained when no PCR product was added to the hybridisation mixture in Luminex assay were considered the background values. The cutoff was computed by adding 5 MFI to 1.1× the median background value of that specific HPV probe [5]. After every plate was read, the cutoff was calculated to determine the HPV genotype presence.

## Results

**Figure 1.**
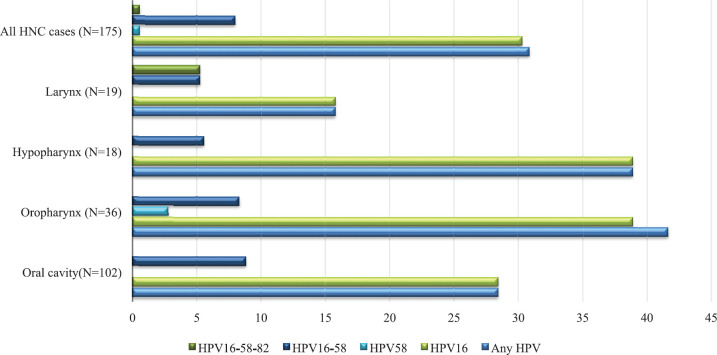
HPV and its genotypes distribution in the study. HPV: Human papillomavirus; HNC: head and neck cancer. All samples were negative for HPV6, HPV11, HPV18, HPV26, HPV31, HPV33, HPV35, HPV39, HPV45, HPV51, HPV52, HPV53, HPV56, HPV59, HPV66, HPV68, HPV70 and HPV73.

Table 1 summarises the characteristics of the study population. HPV-positive cases belonged to younger age groups as compared to HPV-negative HNC cases. They had a lower proportion of tobacco chewers, higher proportion of tobacco smokers and alcohol drinkers, who were HPV-negative participants. The highest HPV-positive cases were from the North, followed by West, East, Central and South regions. Table 2 reports the HPV prevalence observed in the study. The overall HPV prevalence in HNC was 30.86%; the prevalence sub-site wise is OC = 28.43%, OPC = 41.67%, HPC = 38.89% and LC = 15.79% as depicted in Figure 1. The most common HPV genotype was HPV16 in all HNC sub-sites. HPV16 prevalence was highest in OPC and HPC (38.89%). We also found HPV58 in OPC (2.78%). HPV genotype co-infections were also observed. HPV co-infections of HPV16/HPV58 and HPV16/HPV58/HPV82 were observed. HPV16/HPV58 co-infection was observed in all HNC sub-sites, most prevalent in OPC (11.11%). HPV16/HPV58/HPV82 co-infection was observed in LC only (5.26%) (Table 2).

## Discussion

In this prevalence study, we examined the presence of 21 genotypes of HPV in HNC from fresh frozen tumour tissue. Fresh tumour tissues are relatively better biological specimens than serum or plasma to analyse current HPV infection. In our study, we used a type-specific multiplex HPV genotyping (E7-MPG) assay, which combines multiplex PCR and bead-based Luminex technology (Luminex Corporation, Austin, TX) [5]. A comparative study observed E7-MPG assay to have better analytical sensitivity than GP5+/6+-based PCR, linear array and hybrid capture 2 to detect HPV DNA [6–8]. The total number of new HNC cases treated at our institution was 2,597 in 2018.

A recent study by Gheit *et al* [8] conducted in central India determined HPV DNA, RNA and p16^INK4a^ in retrospectively collected paraffin-embedded tumour tissue specimens. They found HPV DNA positivity of 13.7% in the overall HNCs; HPV16 positivity was 72% in all DNA positive tumours. However, the study concluded that p16^INK4a^ is not a good surrogate marker of HPV transformation in Indian HNC cases which is in contrast to European studies. The differences in the prevalence in our study might be due to Tata Memorial Hospital being a referral centre which receives a heterogeneous pool of patients from all geographical regions of India.

In this study, of all HPV HNC positives (76.8%) we found independently positive for HPV16 DNA. The presence of HPV16 DNA, although necessary, does not establish causality as it might be due to transient infections. Our study also found multiple co-infections of HPV16/HPV58 and HPV16/HPV58/HPV82, which have never been reported in the Indian population [9]. However, these findings need to be validated on a larger sample size. HPV-positive individuals had a higher proportion of tobacco and alcohol users which suggests that development of HNC may not be attributed to HPV alone, but possibly to its synergetic effect with tobacco.

A comparison of the prevalence studies on HPV in India in development of the HNC tumour is difficult, because of different methods used in HPV detection, type of specimen used and geographical location of the study. Indian studies have reported varying HPV prevalence; these differences may be due to different detecting methodologies or demographics [10–13]. Our study shows a high prevalence of HPV16 for all sub-sites of HNC. It will be important to investigate if these infections are active by conducting mRNA expression analysis. HPV16 has been found to be most prevalent in prevalence studies carried out in European and Latin American countries with heterogeneity in HNC subtype prevalence owing to other contributing factors in aetiology of HNC [14–16]. Future studies can be carried out to study HPV prevalence and its role in nasopharyngeal carcinoma. There is an emerging evidence of HPV co-infections (e.g., Epstein Barr Virus) and their role in HNC [17–19, p. 7]. Further studies on larger samples stratified by geographical regions are required to understand the role of HPV in HNC among the Indian population.

## Conclusion

By performing type-specific multiplex PCR HPV genotyping assay on 175 fresh frozen tumour tissue samples, we found HPV16 to be the most prevalent genotype in our study. Out of all the HNC subtypes examined, OPC (41.67%) had the highest prevalence of HPV, followed by HPC, OC and LC. A large collaborative study with uniform standardised methodology is required to understand the role of HPV in different subtypes of HNC in the Indian population.

## Conflicts of interest

Where authors are identified as personnel of the International Agency for Research on Cancer/World Health Organisation, the authors alone are responsible for the views expressed in this article and they do not necessarily represent the decisions, policy or views of the International Agency for Research on Cancer/World Health Organisation.

## Funding

The study was funded by Indian Council of Medical Research grant number ICMR/EU/13/2012/NCD-III.

## Consent to participate

Informed consent was taken from all participants to participate in the study.

## Consent to publish

Informed consent was taken from all participants to disseminate the results of the study.

## Ethics approval

Informed consent was taken from all study participants before the interview and ethical clearance was obtained from the Tata Memorial Hospital Institutional Review Board.

## Availability of data and material

Data will be made available on reasonable request.

## Figures and Tables

**Table 1.** Characteristics of the study population.

Parameters	Categories	HPV; *N* = 175			
		Positive(*N* = 54)		Negative(*N* = 121)	
		Number	%	Number	%
Age at enrolment	18–29	4	7.4	7	5.78
	30–39	5	9.25	22	18.18
	40–49	15	27.7	18	14.87
	50–59	16	29.6	36	29.75
	60–69	12	22.2	27	22.31
	70–79	1	1.85	11	9.09
	80–89	1	1.85	0	0
	Missing	0	0	0	0
	Mean (±SD)	52.26 (±12.07)		51.33 (±13.41)	
Region of residence at enrolment	North	24	44.44	32	26.44
	West	18	33.33	57	47.1
	South	1	1.85	1	0.82
	East	6	11.11	20	16.52
	Central	4	7.4	10	8.26
	Missing	1	1.85	1	0.82
Gender	Males	49	90.7	99	81.1
	Females	5	9.25	22	18.1
	Missing	0	0	0	0
Tobacco chewing	Never	13	24.07	32	26.4
	Ever	37	68.5	82	67.7
	Missing	4	7.4	7	5.78
Tobacco smoking	Never	27	50	74	61.1
	Ever	22	40.74	40	33.05
	Missing	5	9.25	7	5.78
Alcohol drinking	Never	34	62.96	82	67.76
	Ever	16	29.62	32	26.4
	Missing	4	7.4	4	3.3

^a^ North: Uttar Pradesh, Bihar, Delhi, Haryana, Himachal Pradesh, Jammu and Kashmir, Punjab, Rajasthan, Chandigarh and Uttarakhand; West: Goa, Gujarat, Daman and Diu, Dadra and Nagar Haveli and Maharashtra; South: Andhra Pradesh, Karnataka, Kerala, Lakshadweep, Andaman and Nicobar, Tamil Nadu and Telangana; East: Arunachal Pradesh, Assam, Meghalaya, Nagaland, Manipur, Tripura, West Bengal, Jharkhand and Orissa; and Central: Madhya Pradesh and Chhattisgarh

**Table 2.** Prevalence of HPV and its genotypes in HNC tissues.

Parameter	Categories	OC (*N* = 102)			OPC (*N* = 36)			HPC (*N* = 18)			LC (*N* = 19)			All HNC cases (*N* = 175)		
		No.	%	95% CI^a^	No.	%	95% CI^a^	No.	%	95% CI^a^	No.	%	95% CI^a^	No.	%	95% CI^a^
Any HPV^a^		29	28.43	0.20–0.38	15	41.67	0.26–0.58	7	38.89	0.18–0.64	3	15.79	0.04–0.42	54	30.86	0.24–0.38
HPV genotype independent	HPV16	29	28.43	0.20–0.38	14	38.89	0.23–0.56	7	38.89	0.18–0.64	3	15.79	0.04–0.42	53	30.29	0.23–0.37
	HPV58	0	0.00	0.00	1	2.78	0.003–0.18	0	0.00	0.00	0	0.00	0.00	1	0.57	0.0007–0.04
HPV genotype co-infection	HPV16 &58	9	8.82	0.04–0.16	3	8.33	0.02–0.23	1	5.56	0.006–0.35	1	5.26	0.006–0.33	14	8.00	0.04–0.13
	HPV16 & 58 & 82	0	0.00	0.00	0	0.00	0.00	0	0.00	0.00	1	5.26	0.006–0.33	1	0.57	0.0007–0.04

a HPV: Human papillomavirus; HNC: head and neck cancer; CI: Confidence interval; No.: Number

All samples were negative for HPV6, HPV11, HPV18, HPV26, HPV31, HPV33, HPV35, HPV39, HPV45, HPV51, HPV52, HPV53, HPV56, HPV59, HPV66, HPV68, HPV70 and HPV73
